# Homologous recombination proficiency in ovarian and breast cancer patients

**DOI:** 10.1186/s12885-021-08863-9

**Published:** 2021-10-28

**Authors:** Justin Fortune Creeden, Nisha S. Nanavaty, Katelyn R. Einloth, Cassidy E. Gillman, Laura Stanbery, Danae M. Hamouda, Lance Dworkin, John Nemunaitis

**Affiliations:** 1 Department of Neurosciences, University of Toledo College of Medicine and Life Sciences, Toledo, OH USA; 2 Department of Cancer Biology, University of Toledo College of Medicine and Life Sciences, Toledo, OH USA; 3 Department of Surgery, University of Toledo College of Medicine and Life Sciences, Toledo, OH USA; 4 Department of Medicine, University of Toledo College of Medicine and Life Sciences, Toledo, OH USA; 5 Gradalis, Inc., Carrollton, Carrollton, TX USA

**Keywords:** Ovarian cancer, Breast cancer, PARP inhibitor, Homologous recombination, Homologous recombination deficient, Homologous recombination proficient, BRCA

## Abstract

Homologous recombination and DNA repair are important for genome maintenance. Genetic variations in essential homologous recombination genes, including *BRCA1* and *BRCA2* results in homologous recombination deficiency (HRD) and can be a target for therapeutic strategies including poly (ADP-ribose) polymerase inhibitors (PARPi). However, response is limited in patients who are not HRD, highlighting the need for reliable and robust HRD testing. This manuscript will review *BRCA1/2* function and homologous recombination proficiency in respect to breast and ovarian cancer. The current standard testing methods for HRD will be discussed as well as trials leading to approval of PARPi’s. Finally, standard of care treatment and synthetic lethality will be reviewed.

## Background

**Fig. 1:**
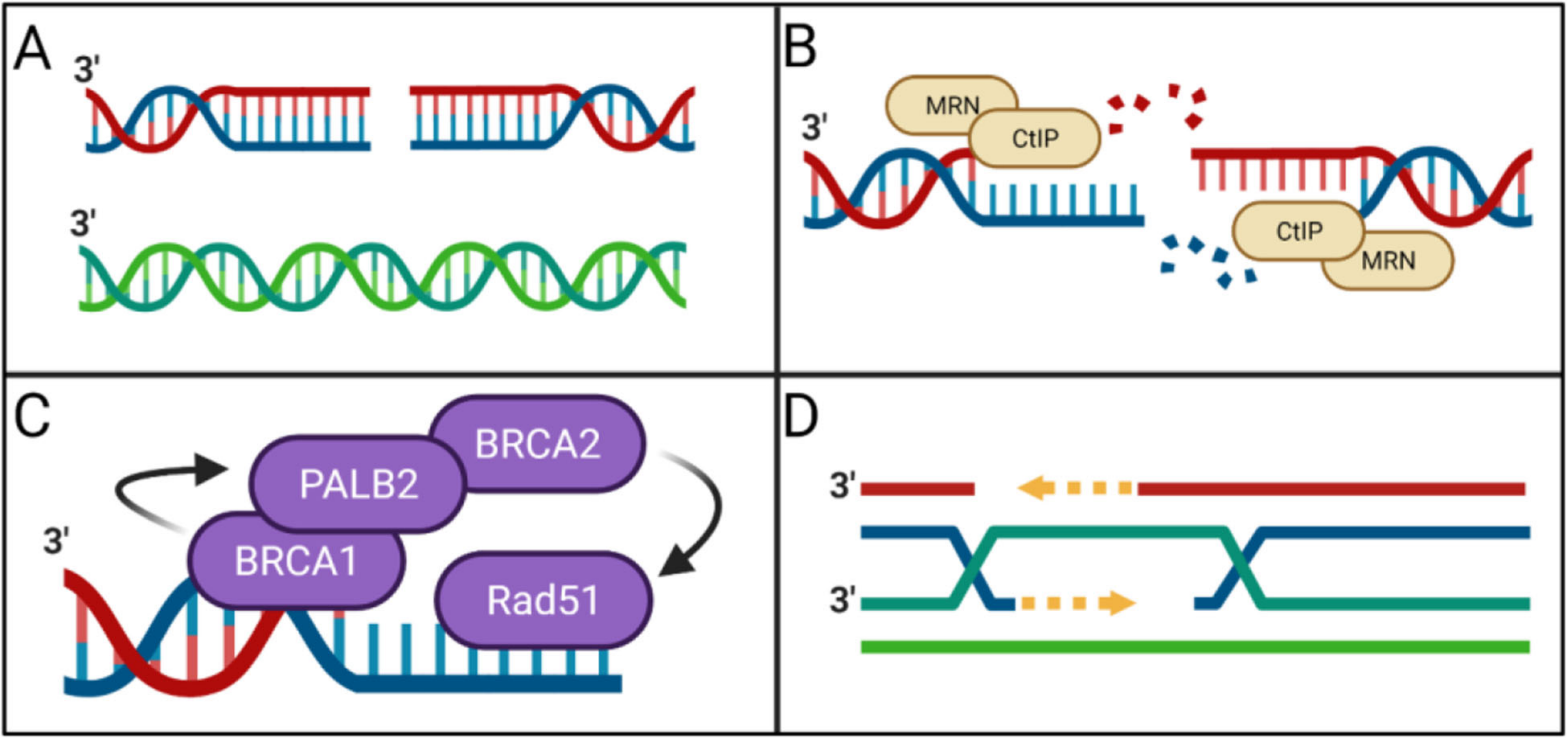
Homologous recombination DNA repair (HRR). **A** double-stranded DNA break and sister chromatid that will be used as a template for repair. **B** MRN and CtIP are involved in DNA resection. **C** HRR repair complex. **D** Schematic representation of BRCA1/2 mediated HRR

DNA damage is inevitable, multifactorial, and dangerous. Whether initiated by exogenous or endogenous sources, inappropriate alterations to the human genome may result in far-reaching, pathological consequence unless quickly and accurately corrected. Homologous recombination DNA repair (HRR) is a critically important mechanism by which DNA damage can be corrected. Homologous recombination DNA repair is a process by which double-stranded DNA breaks and interstrand crosslinks use sister chromatid as a template for repair [1] (Fig. 1A). In this way, DNA damage is removed in an error-free fashion [2]. Additionally, during DNA replication, HRR pathways support the recovery of stalled replication forks [3]. Successful HRR depends on several properly functioning proteins, with BRCA1 and BRCA2 proteins playing particularly pronounced roles [4]. BRCA1 is a tumor suppressor protein central to several macromolecular complexes which drive HRR and cell cycle progression [5]. After MRN and CtIP mediated DNA resection (Fig. 1B), BRCA1 travels to sites of double-stranded DNA breaks where it participates in DNA damage signaling and coordinates DNA damage repair [5, 6]. While the role of BRCA1 in HRR is well established, emerging evidence suggests BRCA1 also regulates cellular selection of double-strand break repair pathways. By doing so, BRCA1 may influence a cell’s choice between HRR and non-homologous DNA end joining (NHEJ) DNA double-strand break repair mechanisms [6, 7]. During the synthesis phase of normal cell cycle progression, if DNA becomes damaged, the BRCA1 protein complexes recruit BRCA2 protein complexes to initiate strand invasion and/or homology-directed repair [5, 6, 8] (Fig. 1C, D). BRCA-dependent DNA double-strand break repair mechanisms can compensate for dysfunctional DNA single-strand break repair mechanisms. When DNA single-strand breaks accumulate and are converted to double-strand breaks, HRR can repair DNA lesions and maintain cell viability [9].

**Fig. 2:**
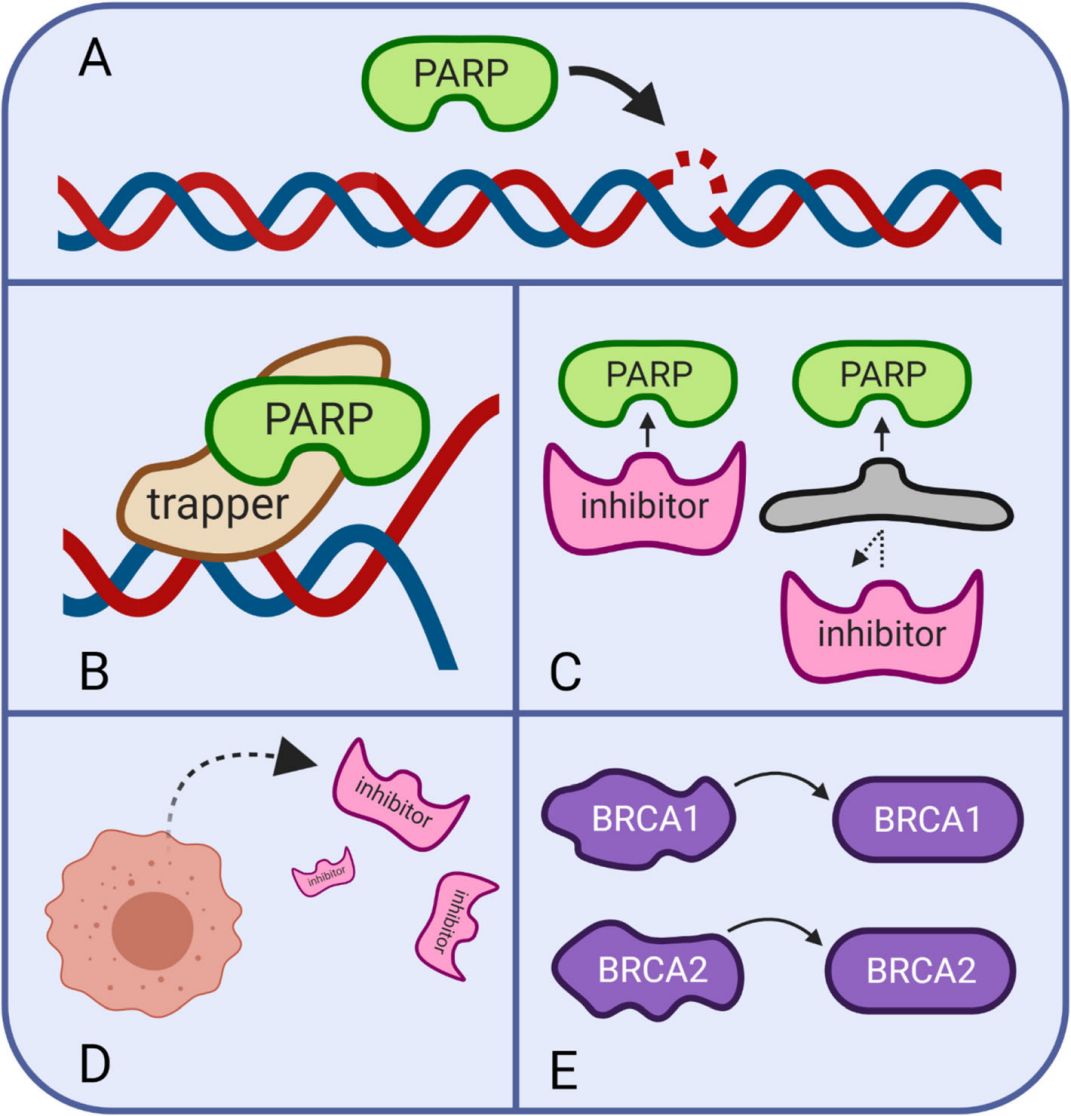
PARP inhibitors (PARPi) and mechanisms of PARPi resistance. **A** PARPs travel to DNA single-strand breaks. **B** PARP inhibitors can trap the enzyme and prevent it from properly functioning. **C**-**E** Potential mechanisms of resistance to PARPi therapy. **C** PARPi may be blocked, **D** effluxed, or **E** reversion of BRCA1/2 mutations

Poly (ADP-ribose) polymerase proteins (PARPs) are nuclear enzymes integral to the base excision repair pathway of single-strand DNA repair [10]. PARPs travel to sites of single-strand DNA breaks (Fig. 2A) where they synthesize polymeric adenosine diphosphate ribose chains for post-translational modification of nuclear proteins [10, 11], in turn promoting downstream single stranded repair processes [12]. Clinically, dysfunctional DNA single-strand break repair may be pharmacologically elicited with PARPis. It is thought that these single-stranded breaks are converted to double-stranded breaks during replication. In cells with *BRCA1/2* mutations resulting in the inability to repair double-stranded breaks, treatment with PAPRis results in synthetic lethality. Beyond its role in single-stranded DNA repair, PARP1 is also involved in the alternative end-joining (alt-EJ) double-strand break repair pathway [13]. PARPs also play a role in homologous recombination, although this role may be relegated to homologous recombination mediated recovery of stalled replication forks, rather than double-stranded break repair [14].

## Main text

### Homologous recombination DNA repair proficiency (HRP) and Cancer

The ability of cancer cells to successfully perform HRR is frequently used as a basis for patient stratification. By grouping patients according HRR status, researchers and clinicians can optimize disease treatments, improve outcome prognostication, and design more informatic clinical trials [15]. Cancer cells that demonstrate homologous recombination DNA repair proficiency (HRP) and cancer cells that demonstrate homologous recombination deficiency (HRD) may be treated using different therapeutic strategies. While HRR status may have important implications for the clinical care of pancreatic cancer patients [16], liver cancer patients [17], lung cancer patients [18], and renal cancer patients [19], its relatively increased incidence in breast and ovarian cancer provides the most robust data regarding treatment effects in this pathway. Indeed, in a sample of 3504 patients with metastatic cancer, genomic footprints indicative of HRD were found in only 6% of cancer cases, while approximately 30% of ovarian cancers and 13% of breast cancers were HRD [20]. In epithelial ovarian cancer (EOC), approximately 50% of cases involve HRD due to alterations of HRR pathway genes [21]. While HRD ovarian cancer cells usually harbor mutations in *BRCA1*, *BRCA2* or other genes with similar features (traits which are collectively referred to as “BRCAness”) [22–24], HRP ovarian cancer cells are often driven by genetic alterations involving other pathways contributing to cell cycle dysregulation, such as cyclin E1 (*CCNE1*) genes [21]. Indeed, *CCNE1* amplification events occur alongside *BRCA* inactivation at remarkably low frequency [25], with Gorski et al. describing *CCNE1* amplification and homologous recombination pathway mutations as “nearly mutually exclusive” [22]. CCNE1 is an important factor in G1/S cell cycle transition, as it activates CDK2 thereby allowing the cell to enter S phase [26]. The amplification of cyclin E1 increases the speed by which cancer cells pass from the G1 to S phase. This can lead to genomic instability and drive the dysregulation of genes responsible for proliferation and cellular survival [22, 27]. Cyclin E1 amplification occurs in 19.1% of all ovarian cancers [22, 25] and 3.4% of breast cancers [28].

### Homologous recombination DNA repair deficiency (HRD) and Cancer

Most HRD cancer cells have direct deficiency in a gene or group of genes responsible for homologous recombination DNA repair, although upregulation of miRNAs can also cause HRD [21]. Generally, the etiology of HRD can be attributed to pathogenic germline variants, somatic mutations, or epigenetic changes in HRR pathway genes. Pathogenic or likely pathogenic germline *BRCA1* or *BRCA2* variants are present in 18% of ovarian cancer cases [29]. In breast cancer, pathogenic or likely pathogenic germline *BRCA1* or *BRCA2* variants are present in 6.1% of all cases [30–36], and 10–20% of triple-negative breast cancer cases [37]. Pathogenic or likely pathogenic germline *BRCA* variants are more often associated with the development of cancer at a younger age and present with more aggressive disease phenotypes with worse prognoses when compared to cancers caused by somatic *BRCA* mutations [38, 39]. In Somatic *BRCA1* and *BRCA2* mutations are identified in 3 % of high-grade serous ovarian carcinoma cases [40]. In ovarian, fallopian tube, and peritoneal carcinomas, the most common somatic mutations in HR genes are *BRCA1* (54% of somatic mutations) and *BRCA2* (17% of somatic mutations) [41]. Somatic mutations of *BRCA1* and *BRCA2* in breast cancer are positively correlated with cancer survival [42–44]. Identification of somatic mutations in cancer is generally associated with a better prognosis then cancers involving pathogenic or likely pathogenic germline variants. Additionally, epigenetic causes of HRD involve the silencing of *BRCA* genes by up or down regulated miRNA activity or *BRCA* promoter hypermethylation [45]. Analogous miR-1255b, miR-148b, and miR-193b miRNA molecules targeting BRCAness genes have been described in ovarian cancer [46]. In breast cancer, miR-182 overexpression induces HRD by targeting *BRCA1* [47]. Furthermore, *BRCA* expression may be inhibited by epigenetic hypermethylation. In 11% of high-grade ovarian carcinomas, *BRCA1* expression is silenced by hypermethylation [40]. Additionally, hypermethylation of *BRCA* promoter regions are associated with more aggressive disease. Hypermethylation of *BRCA1* in ovarian cancers correlate with significantly shorter median survival (*n* = 11, 35.6 months) compared to germline *BRCA1* (*n* = 22, 78.6 months) and wild-type *BRCA1* (*n* = 30, 63.3 months) [40]. HRD etiology may play a critical role in clinical selection of therapeutics as well as overall patient prognosis.

### Role of BRCA1/2 in homologous recombination DNA repair (HRR)

*BRCA1* and *BRCA2* mutations put individuals at higher risk for developing certain malignancies, particularly ovarian and breast cancer. The chance of developing ovarian cancer if an individual has a *BRCA1* mutation is 39–46% [48–52]. In women with a *BRCA1* mutation, the probability of developing breast cancer over her lifetime is 57–65% [50, 53–55]. For *BRCA2* mutations, the chance of developing breast cancer is 45–49%, and for ovarian cancer it is 11–18% [39, 50, 53]. Further, patients demonstrate high prevalence of *BRCA* mutations in triple negative breast cancer (TNBC), which is negative for estrogen receptors (ER-), progesterone receptors (PR-), and excess human epidermal growth factor receptor 2 (HER2) proteins. In fact, 80% of women with a pathogenic mutation in *BRCA1* who develop breast cancer have triple negative disease [56, 57].

Within the genes *BRCA1* or *BRCA2*, cancer cluster regions are genetic regions containing a disproportion amount of gene mutations. A risk hazard ratio (RHR) quantifying the chances of developing cancer is used below to evaluate breast versus ovarian cancer for different cancer cluster regions. In ovarian cancer, there is a single ovarian cancer region: c.1380 to c.4062 (RHR = 0.62; 95% CI, 0.56–0.70; *P* = 9 × 10^− 17^) in *BRCA1* [58]. In breast cancer, there are three breast cancer cluster regions in *BRCA1*: c.179 to c.505 (RHR = 1.46; 95% CI, 1.22–1.74; *P* = 2 × 10^− 6^), c.4328 to c.4945 (RHR = 1.34; 95% CI, 1.01–1.78; *P* = .04), and c.5261 to c.5563 (RHR = 1.38; 95% CI, 1.22–1.55; *P* = 6 × 10^− 9^). There are also three identified breast cancer cluster regions in *BRCA2*: c.1 to c.596 (BCCR1; RHR = 1.71; 95% CI, 1.06–2.78; *P* = .03), c.772 to c.1806 (BCCR13; RHR = 1.63; 95% CI, 1.10–2.40; *P* = .01), and c.7394 to c.8904 (BCCR2; RHR = 2.31; 95% CI, 1.69–3.16; *P* = .00002). The cancer cluster region for *BRCA2* in ovarian cancer is located from c.3249 to c.5681 (RHR = 0.51; 95% CI, 0.44–0.60; *P* = 6 × 10^− 17^) and c.6645 to c.7471 (; RHR = 0.57; 95% CI, 0.41–0.80; *P* = .001) [58]. These breast and ovarian cancer cluster regions are shown below in Table 1. The probability of developing cancer and which type of cancer depends, in part, on the particular cancer cluster region affected within the *BRCA* genes.

**Table 1:** Location of cancer cluster regions in *BRCA1* and *BRCA2* for breast and ovarian cancer [58]

	***BRCA 1***	Exon	Domain	***BRCA 2***	Exon	Domain	
**Breast Cancer Cluster Region**	c.179 to c.505	5	RING	c.1 to c.596	1	–	
	c.4328 to c.4945	13	Serine Cluster	c.772 to c.1806	3	–	
	c.5261 to c.5563	19	BRCT	c.7394 to c.8904	14	DNA binding	
**Ovarian Cancer Cluster Region**	c.1380 to c.4062	11	Part of region contains Serine Cluster	c.3249 to c.5681	11	BRC	
				c.6645 to c.7471	11	BRC	

There are also some regions of *BRCA* that are more commonly mutated in certain populations. The founder mutations, including the *BRCA2* region of c.3249 to c.5681 associated with the c.5946del mutation, is common in patients with Ashkenazi Jewish ancestry [39, 58, 59]. Individuals with this mutation are more prone to develop ovarian cancer than breast cancer. Another founder mutation is the c.5266dupC mutation in *BRCA1*. It is also associated with individuals of Ashkenazi Jewish ancestry as well as European ancestry. This mutation is associated with higher risk of ovarian cancer [39, 60]. The third founder mutation is c.68_69delAG which is located in exon 2 of *BRCA1*; this is more commonly seen in patients with Ashkenazi Jewish ancestry as well as Indian ancestry; which occurs at a frequency of 16.4% in these populations [39]. The mutation is associated with increased sensitivity of ovarian cancer cells to cisplatin therapy, independent of wild-type *BRCA1* alleles [61, 62]. One possible mechanism for this involves the *BRCA* c.68_69delAG mutation increasing expression of maspin, a mammary serine protease inhibitor, as it is a novel downstream target of the BRCA c.68_69delAG mutation [62]. The increased expression of maspin causes a decreased expression of inhibitor of apoptosis proteins [63]. Increased expression of maspin is associated with increased response to cisplatin therapy; as such, it is also associated with a more favorable prognosis in ovarian cancer [39, 62].

Dysfunction in “BRCAness” genes, such as *RAD51* and *CDK12*, can also cause HRD and is an important pathway in the development of ovarian [21] or breast [64] cancer. *CDK12*, which promotes transcription in several HR pathway genes, such as *BRCA1*, is one of the most frequently mutated genes in ovarian cancer [65, 66]. Inactivation of *CDK12* leads to suppression of HRR. Between *BRCA1* exons 11 and 13, there is a binding site for RB1, PALB2, and RAD51 [39, 67]. Interaction between BRCA1 and BRCA2 is mediated by PALB2. This interaction is critical in the RAD51-mediated HRR of damaged DNA [39]. RAD51’s activity is modulated by BRCA2 and the correlation between RAD51 and BRCA2 is important for the repair of double-strand DNA breaks [68].

Homozygous loss of *PTEN* and amplification of *EMSY* may also be involved in HRD, although this is debated as there is not enough information to classify *PTEN* or *EMSY* as a HRD or HRP related defect. EMSY colocalizes with BRCA2 at sites of DNA damage and is located at 11q13 [69]. Most sources do describe *EMSY* amplification or overexpression as a mechanism underlying HRD, although this is controversial and varies from source to source [21]. An *EMSY* amplification is associated with a poor prognosis in breast and ovarian cancer [70]. Amplification or overexpression of the *EMSY* transcriptional repressor leads to *BRCA2* silencing in approximately 20% of high-grade ovarian carcinoma cases [40]. *PTEN* acts as a tumor suppressor gene in its regulation of the cell cycle [71]. It is not clear whether *PTEN* is involved in HRD [21, 41]. *PTEN* deficiency has been shown to be synthetically lethal with PARP inhibition but this may be due to the downregulation of *RAD51*, a gene that assists in the repair of double strand DNA breaks [72, 73]. PARP-1 recognizes DNA breaks and is involved in early recruitment of factors facilitating double-strand break repair, and inhibition of PARP can cause cell death as PARP can no longer recruit factors for cell-repair [74].

#### HRR status determination

##### BRCA testing

Given the significance of *BRCA1/2* gene integrity in HRR functionality, *BRCA1/*2 are often used as a metric for determining a tumor’s HRR status [75–77]. Because the nature of a given *BRCA1/2* variation may predict patient response to certain therapies, it has been shown to be beneficial to differentiate between pathogenic germline variants, somatic mutations, or epigenetic changes. Assessments of pathogenic germline variants require blood or saliva samples, while somatic mutations require direct biopsy or circulating tumor DNA analysis [29]. Epigenetic testing typically relies on formalin-fixed paraffin-embedded (FFPE) tissue samples [78]. However, recent studies are exploring the use of blood samples [79] to identify *BRCA1/2* epigenetic changes, with one such study successfully identifying epigenetic changes using hair, buccal mucosa, and blood samples [80].

For years, Sanger sequencing was the gold-standard to detect single-nucleotide alterations, insertions, and deletions in *BRCA1/2* genetic sequences, while large genomic alterations were detected using multiplex ligation-dependent probe amplification (MLPA) [81]. However, Sanger sequencing and MLPA are time-consuming and costly. Today, samples are typically analyzed using massive parallel sequencing (MPS) [82]. Multiple studies report that using MPS to analyze *BRCA1/2* mutations yields comparable results to Sanger sequencing but on a faster time scale and more cost effective. These factors have contributed to MPS becoming standard practice in recent years [83, 84]. The high-throughput process of MPS allows for the discovery of tiny variants in an individual’s DNA. These variants can be categorized on a spectrum from benign to pathogenic, or they can be categorized as variants of unknown significance (VUS). The identification of VUS results pose a dilemma for both clinicians and patients: additional screening and testing based on a VUS can lead to overtreatment and mismanagement, while patients carrying VUS may experience additional anxiety about potential implications of the variants. In general, known VUS are disclosed to patients, although VUS alone should not change the management of a patient. Attempts are typically made to classify VUS as either benign or pathogenic, although further research and guidelines are needed to determine how best to proceed following the discovery of VUS [85].

Regardless of how mutations are sequenced, the high prevalence of *BRCA1/2* mutations necessitate genetic testing in individuals at risk for ovarian or breast cancer. In high-grade serous ovarian cancer (HGSOC), pathogenic germline variants and somatic *BRCA1/2* mutations can be found in 17–25% of patients, and 18–30% of all *BRCA1/2* variations are somatic in origin [41, 86, 87]. However, germline DNA tests are more sensitive and less invasive than somatic DNA tests, and therefore germline testing is prioritized [21]. However, if germline DNA testing is negative for *BRCA* variants, the American Society of Clinical Oncology (ASCO) guidelines recommend a tumor sample be harvested and tested for somatic mutation [75, 88, 89]. .At the present time, most laboratories do not test for epigenetic changes such as *BRCA* promoter hypermethylation, although there is evidence supporting an improved response to certain therapeutic agents for such mutations [90].

Determining a tumor’s HRR status can aid clinicians and patients in the selection of potential therapeutic strategies, but inconsistencies in laboratory testing procedures detract from their overall utility. Although laboratories across the world have been evaluating *BRCA1/2* genes for over two decades, the extent to which *BRCA1/2* variations are measured, consideration of non-coding DNA regions in genetic sequencing, and which technologies are used to identify large genetic rearrangements all fluctuate drastically between laboratories [82]. Improved institutional guidelines standardizing *BRCA1/2* testing may improve the sensitivity of these tests, particularly in identifying mutations that might not have been discovered without uniform guidelines.

Clinically, *BRCA* testing is vastly underutilized. Only 20% of women eligible for genetic testing based on age at diagnosis and family history per 2017 NCCN guidelines have been tested [79, 91], and it is estimated that over 97% of *BRCA* carriers in the population remain unidentified [80]. Various reasons for low detection rates include small family size, which makes it difficult to identify patterns in hereditary genetic variants, lack of consideration for paternally inherited genetic variants, incomplete penetrance, population migration, limited public awareness about *BRCA*, and poor referral guideline implementation by both primary care providers and oncologists. Other limitations include socioeconomic and geographic factors including limited access to genetic counseling in rural areas, lack of insurance coverage and reimbursement for genetic counseling services, and the time requirement to counsel patients [92–94].

While *BRCA* mutations are more commonly associated with breast and ovarian cancers, men are also affected by the presence of a *BRCA* mutation. Male breast cancer is overall rare, with a lifetime risk of 0.1%. However, the lifetime risk of breast cancer is significantly higher in men with *BRCA1* or *BRCA2* mutation, with a incidence of 1.2% in men who carry a *BRCA1* mutation and 6.8% in men who carry a *BRCA2* mutation [95]. .Men with *BRCA* mutations are also at increased risk for other cancers, such as prostate, pancreatic, and gastric cancers, as well as melanoma of the skin and eye [96]. .By improving and increasing access for *BRCA* testing, men and women alike can be informed about their potential risks of developing cancer.

##### Non-BRCA testing

The need for HRR tests evaluating non-*BRCA1/2* genetic abnormalities is growing with our understanding that HRD may be caused by other dysfunctional “BRCAness” proteins, such as RAD51. Indeed, ovarian and breast cancer patients without *BRCA1/2* mutations demonstrate positive, yet variable, clinical responses to therapeutic agents that target HRD [25]. This supports non-*BRCA1/2* etiologies for HRD and suggests that identification of non-*BRCA1/2* etiologies may provide relevant information for therapeutic selection and may thereby impact clinical prognosis. Improved laboratory testing procedures which evaluate other genes implicated in HRR could result in the development of more personalized, comprehensive treatment plans for patients with cancer. *RAD51*, for instance, is critically involved in HRR processes [97]. *RAD51* mutations are implicated in the development and progression of ovarian and breast cancer [98–100]. Mutational analysis of *RAD51* in 125 families from 12 countries across Europe and North American found an association between *RAD51C/D* mutations and increased risk of ovarian cancer (RAD51C: *p* < 0.001, RAD51D: *p* < 0.001) [101]. Pre-clinical models also suggest *RAD51* mutations may be associated with resistance to anti-cancer therapeutics that target the HRR pathway [102]. Data such as these suggest that pathogenic germline *RAD51* variants may be an effective biomarker for HRD. Accordingly, many, but not all, breast and ovarian cancer panels include assessment of *RAD51* [103]. Inclusion of genes such as *RAD51* in HRR testing procedures may increase detection of HRD in patients without *BRCA1/2* mutations [104] and act as a determinant of therapeutic efficacy and prognosis [105].

##### Other testing strategies

As an alternative to gene-specific testing practices, HRR can also be evaluated by measuring genomic scarring—broadly defined as genomic aberrations of a known origin [106]. More specifically, genomic scarring can be defined here as HRD-related genomic aberrations or large-scale DNA alterations [107]. These scars are associated with unrepaired damage within a patient’s genome resulting from an inability to successfully repair double-strand breaks [107]. This method is used by Myriad in the MyChoice® CDx assay, which is approved as a companion diagnostic for ovarian cancer treatment with olaparib and niraparib. MyChoice® CDx evaluates genomic scarring by measuring loss of heterozygosity (LOH), telomeric allelic imbalances, and large-scale state transitions. A tumor is considered HRD if there is a *BRCA1/2* mutation, or a genomic instability score (GIS) of ≥42. In the QUADRA trial, patients with recurrent platinum sensitive HRD ovarian cancer who had undergone at least 3 lines of prior therapy without prior exposure to PARP inhibitor demonstrated a significant response to niraparib (ORR 29%; 95% CI 16–44; *p* = 0.0003) [108]. Patients with ovarian cancer who received frontline niraparib also demonstrated an improved PFS of 21.9 months versus 10.4 months (HR = 0.43; 95% CI 0.50–0.76; *p* = < 0.001) for tumors which were HRD. However, veliparib, another PARP inhibitor which was evaluated in combination with chemotherapy in first-line ovarian cancer, used a threshold score of ≥33 after retrospective analysis of this cutoff in triple negative breast cancer, and demonstrated increased sensitivity [109, 110]. When veliparib is combined with first line chemotherapy followed by veliparib maintenance, patients demonstrated a progression free survival benefit of 31.9 versus 20.5 months (HR 0.57; 95% CI 0.43–0.76; *p* = < 0.001) in the HRD ≥33 cohort [109].

Without focusing on a specific gene, these metrics approximate the burden caused by dysfunctional repair pathways and allow indirect identification of HRD. Therefore, choosing a cutoff may be therapy- or assay-dependent. For instance, Foundation Medicine identifies HRD through mutation in *BRCA1/*2 or an LOH score of > 16. While Foundation Medicine is not an FDA-approved companion diagnostic for PARPi, it may be used to inform decision making.

Genomic scarring acts as an objective indicator of genomic abnormalities, compared to gene mutations which can be influenced by a variety of factors. Thus, genomic scar biomarkers have strong negative predictive value (NPV) for response to HR-deficiency therapies--meaning individuals without genomic scarring biomarkers will likely not benefit from HR deficiency-targeting drugs. However, they are also poor positive predictive value (PPV) biomarkers, as high levels of genomic instability do not account for mutations that may restore HR proficiency [106]. While genomic scarring has been used to predict HRD in conjunction with other companion diagnostics, improved screening methods and appropriate definition of HRD positivity using genomic scarring represent an active area of research.

#### Homologous recombination ability and cancer therapeutics

##### Standard of care for breast and ovarian cancer

Improved mechanistic understanding and higher resolution laboratory identification of *BRCA1/2* genetic variation subtypes and non-*BRCA1/2* genetic aberrations contribute to the clinical care that breast and ovarian cancer patients receive. In fact, of the 5–10% of breast cancer cases that are related to genetic mutations, 67% of those cases are due to *BRCA1/2* mutations [108]. Broadly, the standard of care for breast and ovarian cancer patients depends on many factors. The standard of care for breast cancer depends on classification, receptor status, and whether it has become invasive or metastatic. Based on these factors, recommendations for breast cancer patients may include a surgical excision and lymph node evaluation, radiation and medical therapy [111]. The standard of care for patients with epithelial ovarian cancer includes maximal surgical cytoreduction and systemic platinum-based chemotherapy [112]. The chemotherapeutic agent and whether chemotherapy is undergone prior to surgery are both determined by the stage and histology of the tumor [113] Stage IIIC and IV ovarian cancers are treated with chemotherapy, either after surgery or before, as neoadjuvant therapy [114]. In both diseases, medical therapy is almost always initiated [111, 113]. However, clinical selection of chemotherapeutic agents depends on various factors, one of which being the HRR status of the cancer cells.

Medical therapyis an important component of breast and ovarian cancer management.. Because HRD cancer cells are more sensitive to certain anti-cancer drugs, such as platinum chemotherapy, the HRR status of a patient’s tumor may influence chemotherapeutic selection. Past research has shown that ovarian cancer patients with *BRCA* mutations are more susceptible to platinum-based chemotherapeutic agents. These agents, such as carboplatin, damage DNA and induce double-strand breaks, which HRD cancer cells cannot repair, which lead to apoptosis [115]. However, platinum-based agents are not without their drawbacks. These agents are associated with significant neurotoxicity, ematogenecity, and marrow suppression which can impact a patient’s quality of life [116]. Like platinum-based agents, other chemotherapeutic agents also function by inducing double-strand breaks, whether it be directly, like doxorubicin, or by crosslinking DNA through alkylation, like cyclophosphamide. One study involving triple negative breast cancer demonstrated that patients with HRD biomarkers were more susceptible to adjuvant doxorubicin and cyclophosphamide combination therapy. Moreover, the HRD patients undergoing the combination chemotherapy demonstrated better disease-free survival than those who were not HRD [117]. .Given evidence of HRD cancers manifesting increased sensitivity to chemotherapy agents that target malignant cell defective repair mechanisms, the identification of biomarkers for HRD could lead to more effective treatment for this subset of patients.

While PARP inhibition has shown promising results in the treatment of HRD cancers, more research is needed in order to establish optimal treatment regimens for HRP cancers. Gemogenovatucel-T, or Vigil, is a vaccine composed of autologous tumor cells, transfected with a plasmid containing GM-CSF and bi-shRNA to decrease furin activity. Decreased furin expression subsequently down-regulates *TGF-β1* and *TGF-β2* expression [118]. Safety and efficacy of Vigil has been demonstrated in numerous solid tumors [119–123]. A recent study exploring the efficacy of gemogenovatucel-T in ovarian cancer patients demonstrated significant differences in RFS and OS in patients with *BRCA* wild-type tumors when compared to those who had *BRCA* mutations [124]. Subsequent analysis of patients who were HRP versus HRD revealed further RFS (10.6 vs 5.7 months; HR = 0.386 90% CI 0.199–0.750 *p* = 0.007) and OS (NR vs 26.9 months; HR = 0.342 90% CI 0.141–0.832 *p* = 0.019) benefit [125]. To compare, *BRCA*-wt/HRP patients treated with niraparib who had a response to first line chemotherapy in the PRIMA trial had a PFS of 8.1 versus 5.4 months for placebo (HR 0.68 95% CI 0.49–0.94 *p* = 0.020) [126]. Patients with HRP or unknown HR status treated with olaparib and bevacizumab as first line maintenance had a median PFS of 16.9 months vs 16.0 months in placebo treated (HR 0.92 95% CI 0.72 to 1.17) [77]. Vigil demonstrated improved clinical benefit compared to niraparib or olaparib and bevacizumab with no grade 3/4 adverse events reported in patients receiving Vigil. Both niraparib and olaparib plus bevacizumab result in a large amount of drug related grade 3/4 adverse events (65.3 and 57.0% respectively) and dose discontinuation (14.7 and 41.0% respectively) [77, 126]. Additionally, there has been some concern regarding the rate of treatment related myelodysplastic syndrome and acute myeloid leukemia (tMDS/AML) following PARPi. In a recent meta-analysis, risk of tMDS/AML was increased in patients receiving PARPi compared to placebo (Peto OR 2.63 95% CI 1.13–6.14; *p* = 0.026) [127]. Prognosis for tMDS/AML is typically poor, with a 5 year survival of less than 10% [110]. Therefore, Vigil is an attractive therapeutic option for frontline ovarian cancer maintenance with improved efficacy and robust safety.

The mechanism for Vigil benefit in HRP patients remains unclear; however, the level of clonal neoantigens present in cells that are capable of homologous recombination may play a role. Colon cancer tumors with mismatch repair deficiency (MMR) have a high proportion of neoantigens. Neoantigens have, therefore, been investigated as a predictive biomarker for response to immunotherapies [128, 129]. However, the amount of neoantigens present in a tumor may not be the sole predictor of response to immunotherapies, and may differ based on immunotherapy mechanisms. Activated and primed T cells may not recognize all neoantigens with the same affinity. McGranahan et al. found that T cells recognize clonal neoantigens compared to subclonal neoantigens preferentially to target the tumor [130]. Vigil has also shown the ability to increase circulating CD3+/CD8+ T cells in advanced cancer patients [131]. Likely these CD3+/CD8 + T cells have been primed to the relevant clonal neoantigens present in higher quantity and concentration in HRP tumors. Therefore, tumors that are HRP may have more clonal neoantigens and may derive clinical benefit from immune based therapies such as vaccinations which increase the primed CD8+ T cell population.

##### Synthetic lethality

Research shows that identification of HRD tumors leads to more effective chemotherapeutic regimens for this subset of patients. Moreover, the understanding of the molecular mechanisms by which HRD cells are defective also gives way to the use of drugs which exploit the phenomenon of synthetic lethality. In this context, synthetic lethality refers to situations in which a single genetic aberration or chemical perturbagen is individually tolerated by a cell, but becomes lethal when combined with another genetic aberration or chemical perturbagen [132]. First described by Bridges in 1922 [133, 134], synthetic lethality now serves as the basis of pharmacological strategies targeting HRD tumor cells. Synthetic lethality may be pharmacologically realized in a number of ways. One of the most well studied involves the genetic aberrations that drive HRD, and PARP inhibitors that suppress single strand break repair mechanisms. While PARP functions in single-strand break repair, homologous recombination repairs double-strand breaks. Therefore, by inhibiting both mechanisms, the cancer cell is effectively unable to repair DNA damage, which then leads to apoptosis [135]. The therapeutic implications of successful HRD-PARPi synthetic lethality reinforces the need for enhanced definition of HRD biomarkers.

PARP inhibitors are being studied and are currently approved for used in the management of breast and ovarian cancer. There are multiple PARP inhibitors that have been approved by FDA for use in cancer treatment, including olaparib, niraparib, rucaparib, and talazoparib. Three of the four drugs, olaparib, rucaparib, and niraparib, are approved for the treatment of ovarian cancer. PARP inhibitors, such as the ones mentioned above, function by way of PARP-trapping. The PARP inhibitors can act on both PARP1 and PARP2 at the location of DNA damage, effectively trapping the enzymes from functioning (Fig. 2B). Since the PARP enzyme is non-functional, it can no longer recruit any other enzymes to repair the damage, and cell death ensues. The trapping mechanism makes PARP inhibition more effective than a knockout of the PARP enzyme because the PARP1-DNA complex demonstrates more cytotoxicity than the original unrepaired single-strand break [136]. Resistance to PARP inhibitors may develop via a number of mechanisms [56, 137] (Fig. 2C, D, E).

While the therapeutic value of PARP inhibitors is often determined based on *BRCA1/2* mutational status, other clinically important tumor suppressor genes also contribute to synthetic lethality. One such example is *RAD51*, another enzyme involved in double-strand break repair. BRCA2 signals RAD51 to travel to sites of DSBs. At these sites, RAD51 will eventually signal strand invasion and subsequent homologous strand exchange for successful damage repair. A study combining the PARP inhibitor olaparib with BRCA2-RAD51 disruption showed synthetic lethality [138]. Another clinically significant relationship is that of DNA-PK, an enzyme that has an essential role in non-homologous end joining. When the subunit Ku on DNA-PK binds to double-strand breaks, it recruits a host of NHEJ proteins that can function to repair the DNA. The combined inactivation of DNA-PK and BRCA1 also results in synthetic lethality. Research has shown that the DNA-PK inhibitor, AZD7648, used in combination with olaparib, leads to cell death [139]. The synergistic effect between so many of these genes and the BRCA proteins expands the possibility of ovarian and breast cancer combination therapies.

## Conclusions

Proficiency or deficiency in HRR is a critical metric of therapeutic selection and prognosis for ovarian and breast cancer patients. Techniques for determining HRR status in patients are currently underutilized, inconsistently implemented, and produce results that are often reduced to binary HRD or HRP designations in clinical practice. The designation of a patient’s tumor for individual HRR status assessment is important because therapeutic efficacy or patient prognosis vary according to the identification of genetic variations (e.g. *BRCA1/2*; *RAD51*, *CDK12*), the nature of these variations (i.e. pathogenic germline variant, somatic mutation, epigenetic change), and the site of these mutations (e.g. c.179 to c.505, c. 4328 to c. 4945). Optimal clinical outcomes require testing which consistently generate these data, and careful consideration of each patient’s tumor’s unique HRR status and etiology. Evidence of therapeutic impact based on HRR status are established in ovarian cancer and likely will have further impact in several other solid tumors.

## Data Availability

Not applicable.

## Abbreviations

HRD: Homologous recombination deficiency
PARP: Poly (ADP-ribose) polymerase
PARPi: Poly (ADP-ribose) polymerase inhibitor
HRR: Homologous recombination DNA repair
HRP: Homologous recombination proficient
NHEJ: Non-homologous DNA end joining
Alt-EJ: Alternative end-joining
EOC: Epithelial ovarian cancer
RHR: Risk hazard ratio
FFPE: Formalin-fixed paraffin-embedded
ASCO: American Society of Clinical Oncology
NPV: Negative predictive value
PPV: Positive predictive value
tMDS: Treatment related myelodysplastic syndrome
tAML: Treatment related acute myeloid leukemia
MMR: Mismatch repair deficiency
DSB: Double stranded break
